# Involvement of STIM1 and Orai1 in EGF-mediated cell growth in retinal pigment epithelial cells

**DOI:** 10.1186/1423-0127-20-41

**Published:** 2013-06-25

**Authors:** I-Hui Yang, Yao-Ting Tsai, Siou-Jin Chiu, Li-Teh Liu, Hsuan-Hung Lee, Ming-Feng Hou, Wen-Li Hsu, Ben-Kuen Chen, Wei-Chiao Chang

**Affiliations:** 1 Department of Medical Genetics, College of Medicine, Kaohsiung Medical University, Kaohsiung, Taiwan; 2 Department of Ophthalmology, Kaohsiung Chang Gung Memorial Hospital and Chang Gung University College of Medicine, Kaohsiung, Taiwan; 3 Department of Medical Laboratory Science and Biotechnology, College of Medicine and Life Science, Chung-Hwa University of Medical Technology, Tainan, Taiwan; 4 Cancer Center, Kaohsiung Medical University Hospital, Kaohsiung, Taiwan; 5 Institute of Clinical Medicine, Kaohsiung Medical University, Kaohsiung, Taiwan; 6 Center for Resources, Research and Development, Kaohsiung Medical University, Kaohsiung, Taiwan; 7 Institute of Bioinformatics and Biosignal Transduction, College of Bioscience and Biotechnology, National Cheng Kung University, Tainan, Taiwan; 8 Department of Clinical Pharmacy, School of Pharmacy, Taipei Medical University, Taipei, Taiwan; 9 Department of Pharmacy, Taipei Medical University-Wanfang Hospital, Taipei, Taiwan; 10 Master Program for Clinical Pharmacogenomics and Pharmacoproteomics, School of Pharmacy, Taipei Medical University, Taipei, Taiwan

**Keywords:** STIM1, Orai1, Store-operated calcium channel, Retinal pigment epithelial cell, Proliferative vitreoretinopathy

## Abstract

**Background:**

In non-excitable cells, one major route for calcium entry is through store-operated calcium (SOC) channels in the plasma membrane. These channels are activated by the emptying of intracellular Ca^2+^ store. STIM1 and Orai1 are major regulators of SOC channels. In this study, we explored the functions of STIM1 and Orai1 in epidermal growth factor (EGF)-induced cell proliferation and migration in retinal pigment epithelial cells (ARPE-19 cell line).

**Results:**

EGF triggers cell proliferation and migration in ARPE-19 cells. Cell proliferation and migration involve STIM1 and Orai1, as well as phosphorylation of extracellular signal-regulated protein kinase (ERK) 1/2, and Akt. Pharmacological inhibitors of SOC channels and siRNA of Orai1 and STIM1 suppress cell proliferation and migration. Pre-treatment of mitogen-activated protein kinase kinase (MEK) inhibitors and a phosphatidylinositol 3 kinases (PI3K) inhibitor attenuated cell proliferation and migration. However, inhibition of the SOC channels failed to prevent EGF-mediated ERK 1/2 and Akt phosphorylation.

**Conclusions:**

Our results showed that STIM1, Orai1, ERK 1/2, and Akt are key determinants of EGF-mediated cell growth in ARPE-19 cells. EGF is a potent growth molecule that has been linked to the development of PVR, and therefore, STIM1, Orai1, as well as the MEK/ERK 1/2 and PI3K/Akt pathways, might be potential therapeutic targets for drugs aimed at treating such disorders.

## Background

The retinal pigment epithelium (RPE) lying between the neurosensory retina and the choroid is vital for normal visual function [1]. Under normal conditions, the RPE cells have limited potential for proliferation and migration. However, in response to trauma, inflammation, or other extracellular stimuli, the RPE cells may proliferate and migrate; this in turn leads to serious vitreoretinal diseases such as proliferative vitreoretinopathy (PVR) [2,3]. PVR is a complication of rhegmatogenous retinal detachment with poor prognosis for visual function. The pathogenesis of PVR comes from the retinal break that exposes the vitreous to the RPE and causes the breakdown of the blood-retinal barrier [4]. The cytokines in the plasma trigger proliferation and migration of the RPE cells, thereby resulting in epiretinal membrane formation, which complicates reattachment surgery [4].

Several lines of evidence have indicated that epidermal growth factor (EGF) is an important regulator in the proliferation and migration of RPE cells during PVR development [5]. For example, expression of EGF was detected in the preretinal membranes, and intravitreal and subretinal fluid cells from PVR patients [6,7]. Recent studies have revealed that co-treatment with EGF and transforming growth factor β1 (TGF-β1) increased the expression of alpha-smooth muscle actin (α-SMA) [8], which was expressed by myofibroblasts in epiretinal membranes from patients with PVR [9]. Therefore, EGF contributes to PVR development.

Previous studies have indicated that EGF can induce calcium signaling via store-operated calcium (SOC) channels [10,11] and subsequent regulation of cell proliferation and migration [12]. Calcium plays an important role in the development and maintenance of many physiological functions [13], and therefore, the intracellular calcium concentration is precisely regulated. An increase in the intracellular calcium concentration results in altered cellular functions such as cell proliferation, cytokines secretion, DNA synthesis, and cell migration [14]. In non-excitable cells, SOC channels are critical modulators of intracellular calcium signaling. Orai1 is a key subunit of SOC channels whereas stromal interaction molecule 1 (STIM1) is a calcium sensor that triggers the activation of SOC channels [15]. Recently, both Orai1 and STIM1 have been identified in RPE cells [14]. However, the roles of STIM1 and Orai1 in the growth of RPE cells are still elusive.

In this study, we hypothesized that STIM1/Orai1-mediated calcium signaling is involved in EGF-induced cell proliferation and migration in ARPE-19 cells. To test this hypothesis, SOC channel inhibitors and small interfering RNA (siRNA) of Orai1 and STIM1 were used to examine the mechanisms of cell proliferation and migration in ARPE-19 cells. The findings of this study provide further insight into the pathogenesis of PVR.

## Methods

### Cell culture

ARPE-19 cells were obtained from the American Type Culture Collection (ATCC) and maintained in Dulbecco’s Modified Eagle’s Medium: F-12 (DMEM:F-12) nutrient mixture (Invitrogen Corp., Carlsbad, CA) with 10% fetal bovine serum (FBS) (Invitrogen Corp., Carlsbad, CA), 100 mg/mL streptomycin and 100 U/mL penicillin (Invitrogen Corp., Carlsbad, CA) at 37°C with 5% CO_2_. In cell proliferation studies, cells were starved with 0.5% FBS in DMEM:F12 for 24 h before EGF treatment.

### WST-1 assay

ARPE-19 cells were pre-treated for 30 min with various inhibitors and then exposed to EGF (25 ng/mL) for 48 h. To assay cell proliferation, cells were incubated with WST-1 reagent (Roche, Indianapolis, IN) at 37°C for 5 to 10 min. A microplate spectrophotometer was used to measure the absorbance of the samples at 450 nm with 600 nm as the reference wavelength.

### Wound healing assay

ARPE-19 cells were seeded in wells of Culture-Insert (Ibidi GmbH, Martinsried, Germany) for 24 h. The Culture-Inserts were removed to reveal the wound gap. The baseline and the wound closure after treatment were photographed at magnification of 100× by a digital camera attached to an inverted microscope (Nikon Eclipse Ti, Nikon, Japan). The photos shown were representative of three independent experiments. For further quantitative analyses, distances between the two edges of the gap in five fields were measured and were used to calculate the relative migration percentage using the following formula: Relative migration percentage = (the distance before migration - the distance after migration)/the distance before migration × 100%.

### Reverse transcription-polymerase chain reaction (RT-PCR)

Total RNA was isolated from ARPE-19 cells with Trizol reagent (Invitrogen Corp., Carlsbad, CA) according to the manufacturer’s instructions. Reverse transcriptase reactions were performed on 1 μg samples of RNA using a reverse transcription kit (Invitrogen Corp., Carlsbad, CA). Incubation conditions included 10 min at 25°C, 120 min at 37°C, and 5 min at 85°C. The resulting cDNAs were used to detect Orai1 and STIM1 expression levels by PCR.

### PCR and DNA gel electrophoresis

After synthesis of cDNA, we used the following gene-specific primers: Orai-1 (196 bp), forward primer: TTC CTA GCT GAG GTG GTG CT; reverse primer: CGA TAA AGA TCA GGC CGA AG; STIM 1 (563 bp), forward primer: GGC AGT ACA CGC CCC AAC CC; reverse primer: CCA GCC AGG TGG GGA ATG CG. After denaturing the DNA at 94°C for 5 min, thirty-five cycles of amplification were performed: 94°C 30 sec, 58°C 30 sec, 72°C 1 min. Then the reaction was incubated at 72°C for 10 min. PCR products were separated by gel electrophoresis in 2% DNA agarose gel using TAE buffer and visualized by ethidium bromide staining and UV transillumination.

### Protein extraction and western blotting

Total cell lysates (40 μg) were isolated in RIPA buffer containing protease inhibitors and analyzed by SDS-PAGE on a 10% gel. After electro-blotting onto polyvinylidene fluoride membranes (Millipore Corp., Bedford, MA), membranes were blocked with 5% non-fat dry milk for 1 h at room temperature. Blots were probed overnight at 4°C with working dilutions of primary antibodies specific for the individual target protein. The dilution of antibody against Orai1 (Santa Cruz Biotechnology, Santa Cruz, CA) was 1:500. The dilution of antibody against STIM1 (BD Transduction Laboratories, Franklin Lakes, NJ) was 1:250. The dilution of antibody against β-actin (Sigma-Aldrich, Saint Louis, MO) was 1:20000. The dilution of antibody against phospho-ERK 1/2 (Millipore Corp., Bedford, MA or Cell Signaling, Beverly, MA) was 1:1000. The dilution of antibody against phospho-Akt (Epitomics, Burlingame, CA) was 1:1000. Membranes were washed three times with 0.1% PBST and incubated with a 1:2000 to 1:10000 dilution of peroxidase-linked anti-rabbit or anti-mouse IgG secondary antibodies (Millipore Corp., Bedford, MA) for 1 h at room temperature. Last, the protein bands were visualized using an ECL-plus Western blotting detection system (Millipore Corp., Bedford, MA).

### Calcium concentration detection

ARPE-19 cells were seeded onto glass coverslips for 24 h. Then the attached cells were loaded with 1 μM Fluo-4 (Invitrogen Corp., Carlsbad, CA) at 37°C for 20 min in the dark. Cells were washed three times in standard external solution: 145 mM NaCl, 2.8 mM KCl, 2 mM CaCl_2_, 2 mM MgCl_2_, 10 mM D-glucose, and 10 mM HEPES, pH 7.4. Changes in fluorescence intensity of Fluo-4 in loaded cells were detected by time-lapse video-microscope (Olympus IX70, Olympus, Japan) and analyzed by the cell^Λ^R system (Olympus, Japan).

### Transfection with siRNA

ARPE-19 cells were seeded for 24 h. Then, the cells were transiently transfected with control siRNA (Santa Cruz Biotechnology, Santa Cruz, CA), Orai1 siRNA (Santa Cruz Biotechnology, Santa Cruz, CA; Applied Biosystems, Foster City, CA), or STIM1 siRNA (Santa Cruz biotechnology, Santa Cruz, CA; Applied Biosystems, Foster City, CA) in Opti-MEM medium (Invitrogen Corp., Carlsbad, CA) containing Lipofectamine™ 2000 (Invitrogen Corp., Carlsbad, CA). In two consecutive days following transfection (transfecting once every 24 h), the cells were treated and prepared for individual experiments.

### Quantitative real-time PCR

The primers used: Orai-1 (196 bp) as previously described; β-actin (145 bp), forward primer: ATC TCC TTC TGC ATC CTG TCG GCA AT; reverse primer: CAT GGA GTC CTG GCA TCC ACG AAA C; and STIM 1 (110 bp), forward primer: AGA AAC ACA CTC TTT GGC ACC; reverse primer: AAT GCT GCT GTC ACC TCG. The SYBR Green PCR master mix reagent (Applied Biosystems, Foster City, CA) was used to amplify the cDNA and the products were detected by Applied Biosystems 7500.

### BrdU (Bromodeoxyuridine) assay

DNA synthesis in proliferating cells was determined by measuring BrdU incorporation with the commercial Cell Proliferation ELISA System (Roche, Indianapolis, IN). ARPE-19 cells were seeded at a density of 3 × 10^3^ per well using 96-well culture plates in 10% FBS with DMEM:F12 for 24 h. For the inhibitor study, the cells were then starved with 0.5% FBS in DMEM:F12 for another 24 h before inhibitors pre-treatment and then EGF (25 ng/mL) treatment for 24 h. For the siRNA study, the cells were transiently transfected with siRNA, and then treated with EGF (25 ng/mL) for 24 h. The cells were incubated for 5 h with BrdU using the kit-supplied 1X BrdU labeling agent (Roche, Indianapolis, IN). The subsequent FixDenat, Anti-BrdU-peroxidase-conjugate, and substrate treatment procedures were performed according to the manufacturer’s instructions. Absorbance values were measured at 405 nm using a microplate spectrophotometer.

### Flow cytometry

ARPE-19 cells were seeded in 10-cm dishes until 80% confluence. Cells were starved for 24 h with 0.5% FBS in DMEM:F12 before inhibitor pre-treatment and then EGF (25 ng/mL) treatment for 24 h. The cells were then harvested by trypsin treatment, collected in 15-mL centrifuge tubes and washed with PBS. The collected cells were fixed at −20°C with 70% ethanol for more than 1 h. After fixation, the ethanol was removed from fixed cells by centrifugation. The fixed cells were then incubated with 1 mL of propidium iodide staining solution for 30 min at room temperature in the dark. The percentage of cells in each phase of the cell cycle was determined by flow cytometry (BD Biosciences, Franklin Lakes, NJ). Approximately 10,000 events (cells) were evaluated for each sample.

### Statistical analysis

All data are shown as means ± SD in figures. Statistical analyses were performed using the Statistical Package for Social Science program (SPSS for Windows, version 13.0; SPSS, Chicago, IL) based on the results of six independent experiments. One-way ANOVA was used for comparing the differences between groups. *P* values less than 0.05 were considered statistically significant.

## Results

### EGF stimulated cell proliferation and migration in ARPE-19 cells

**Figure 1:**
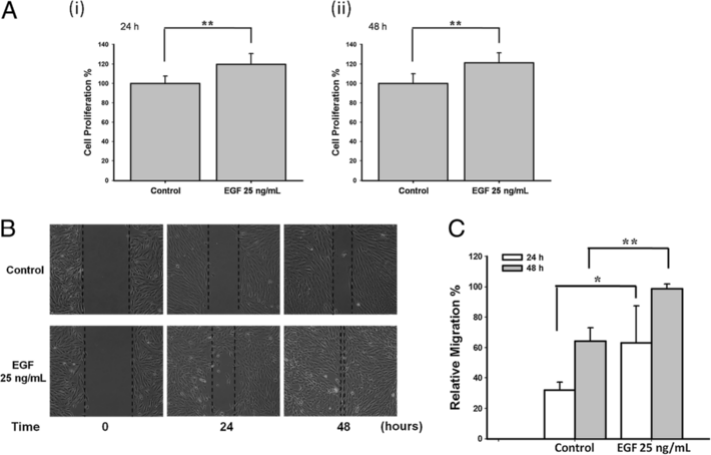
**EGF induced ARPE**-**19 cell proliferation and migration.** (**A**) WST-1 assay was used to test cell proliferation. Cell proliferation of ARPE-19 cells was induced after EGF treatment for 24 h and 48 h (both ** p < 0.01). (**B**) Cell migration was increased after 24 h and 48 h of 25 ng/mL EGF stimulation. (**C**) The quantitative analysis of Figure 1B revealed significant cell migration induced by the treatment of EGF (* p < 0.05 and ** p < 0.01, respectively).

First, we assessed the effects of EGF on ARPE-19 cell proliferation and migration by WST-1 assay and wound healing assay, respectively. Statistically significant increases in cell proliferation were observed following 24 h and 48 h stimulation with 25 ng/mL of EGF (both **p < 0.01; Figure 1A). Cell migrations following 24 h and 48 h stimulation with 25 ng/mL EGF comparing to control were shown in Figure 1B. The quantifications of cell migration were shown in Figure 1C.

### Calcium chelators reduced the EGF-mediated cell proliferation and migration in the ARPE-19 cells

**Figure 2:**
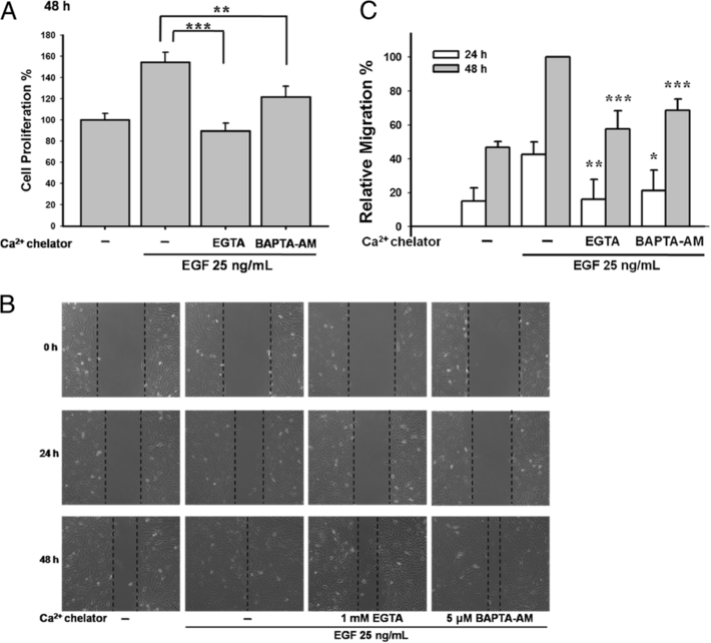
**Calcium chelators reduced the EGF**-**mediated cell proliferation and migration in the ARPE**-**19 cells.** (**A**) Pre-treatment of EGTA (1 mM) or BAPTA-AM (2.5 μM) inhibited EGF-stimulated cell proliferation (*** p < 0.001 and ** p < 0.01, respectively) by WST-1 assay. (**B**) EGTA (1 mM) and BAPTA-AM (5 μM) suppressed EGF-mediated ARPE-19 cell migration. (**C**) The quantitative analysis of Figure 2B showed the statistical significance of suppression in EGF-mediated cell migration by EGTA and BAPTA-AM.

We next used calcium chelators to clarify the involvement of calcium signaling in EGF-mediated cell growth. As shown in Figure 2A, both 1 mM EGTA and 2.5 μM BAPTA-AM significantly inhibited cell proliferation (***p < 0.001 and **p < 0.01, respectively). In addition, Figure 2B and 2C demonstrated that EGTA and BAPTA-AM suppressed cell migration.

### Expression of STIM1/Orai1 and functional SOC in ARPE-19 cells

**Figure 3:**
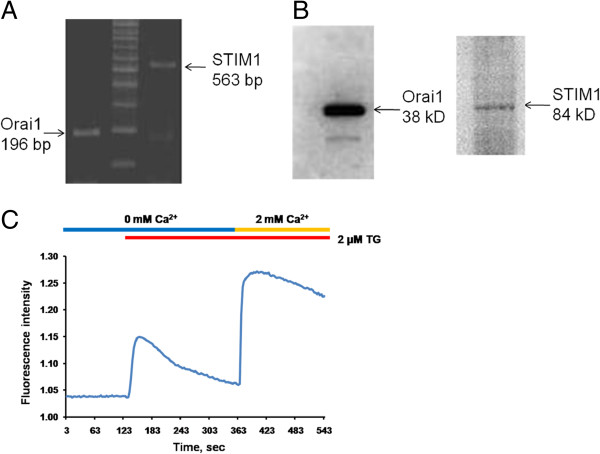
**The expression of STIM1 and Orai1 in ARPE**-**19 cells.** (**A**, **B**) Expression of Orai1 and STIM1 was determined by RT-PCR (**A**) and Western blots (**B**) in ARPE-19 cells. (**C**) **F**luorescent-based calcium assay was used to detect calcium signals. ARPE-19 cells were incubated in calcium free condition with 2 μM thapsigargin (TG). And then 2 mM calcium solution was added to detect the classical SOC entry.

RT-PCR and western blot analysis were used to confirm the existence of Orai1 and STIM1 in the ARPE-19 cells (Figure 3A and B). SOC signals were detected by a classical calcium add-back protocol. Calcium stores were depleted by 2 μM thapsigargin (TG). Calcium influx was observed in the ARPE-19 cells by the addition of 2 mM calcium (Figure 3C).

### The SOC channel inhibitor 2-APB inhibited EGF-mediated cell proliferation and migration

**Figure 4:**
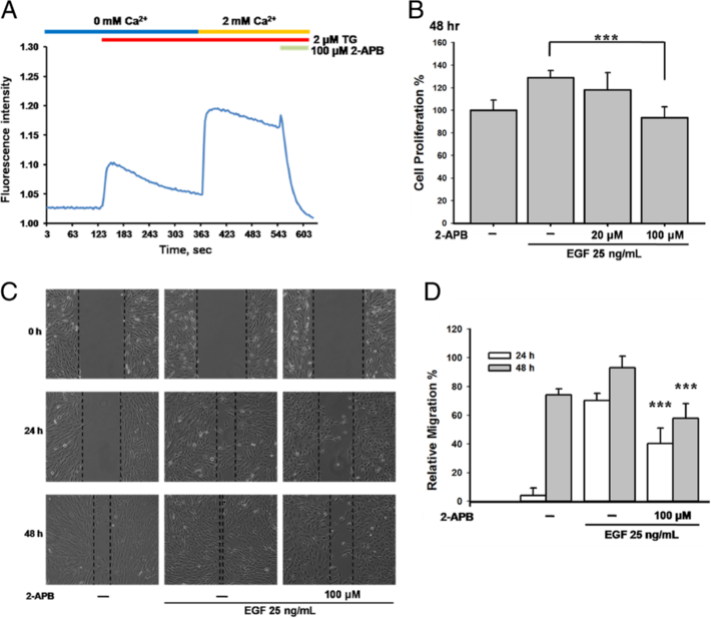
**The inhibitor of SOC channels inhibited EGF**-**mediated cell proliferation and migration in ARPE**-**19 cells.** (**A**) SOC influx evoked by 2 μM TG was suppressed by adding 100 μM 2-APB in ARPE-19 cells. (**B**) ARPE-19 cells were pre-treated with 100 μM 2-APB for 30 min and then were incubated with 25 ng/mL EGF for 48 h. WST-1 assay was used in this study. (**C**) ARPE-19 cells were pre-treated with 100 μM 2-APB for 30 min, and were stimulated by 25 ng/mL EGF for 24 h and 48 h. Wound healing assay was used in this study. (**D**) The quantitative analysis of Figure 4C showed the statistical significance of reduction in EGF-mediated cell migration by 2-APB at 24 h and 48 h (both *** p < 0.001).

2-APB has been widely used to inhibit SOC channels. In ARPE-19 cells, 2 μM TG evoked calcium influx, and the addition of 100 μM 2-APB blocked the calcium signals (Figure 4A), thereby indicating that 2-APB is a reliable inhibitor of SOC channels. We then pre-treated ARPE-19 cells with 20–100 μM 2-APB for 30 min, followed by incubation with 25 ng/mL EGF for 48 h. As shown in Figure 4B, 100 μM 2-APB significantly inhibited the EGF-mediated cell proliferation (***p < 0.001). In addition, 100 μM 2-APB blocked the EGF-mediated cell migration (Figure 4C and 4D).

### Knocking down Orai1 and STIM1 reduced the EGF-mediated cell proliferation and migration

**Figure 5:**
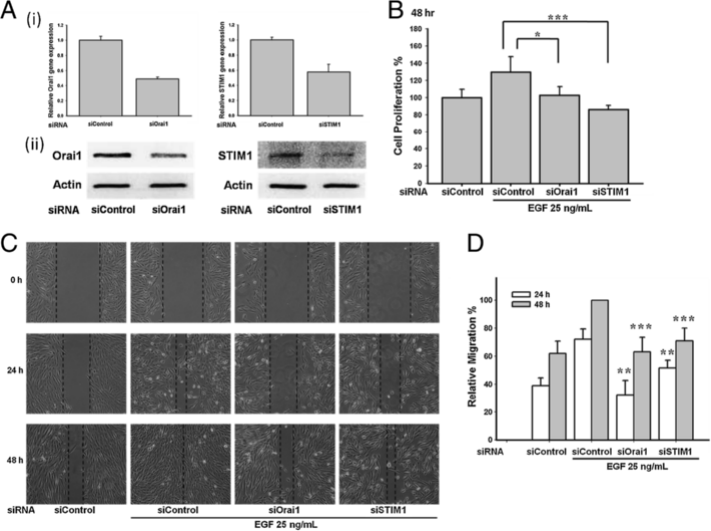
**Knockdown of Orai1 and STIM1 reduced the EGF**-**mediated cell proliferation and migration in ARPE**-**19 cells.** (**A i**, **ii**) ARPE-19 cells were transfected with Orai1 and STIM1 siRNA. Expression of Orai1 and STIM1 was detected by real-time PCR (**i**) and Western blots (**ii**). (**B**-**C**) ARPE-19 cells transfected with Orai1 and STIM1 siRNA suppressed the 25 ng/mL EGF-induced cell proliferation and migration. WST-1 assay or cell migration assay was used in the study. (**D**) Quantitative analysis of cell migration of Figure 5C.

To further confirm the role of STIM1/Orai1 signaling in ARPE-19 cells, Orai1 siRNA and STIM1 siRNA were transfected into the ARPE-19 cells. Orai1 is one of the major subunits of SOC channels and STIM1 is the calcium sensor that triggers the activation of SOC entry. The Orai1 and STIM1 siRNAs reduced expression of their respective mRNA (Figure 5A(i)) and protein (Figure 5A(ii)). Importantly, knocking down Orai1 and STIM1 suppressed cell proliferation (*p < 0.05 and ***p < 0.001, respectively; Figure 5B) and migration (Figure 5C and 5D).

### Role of STIM1/Orai1 in EGF-mediated BrdU incorporation and cell-cycle progression

**Figure 6:**
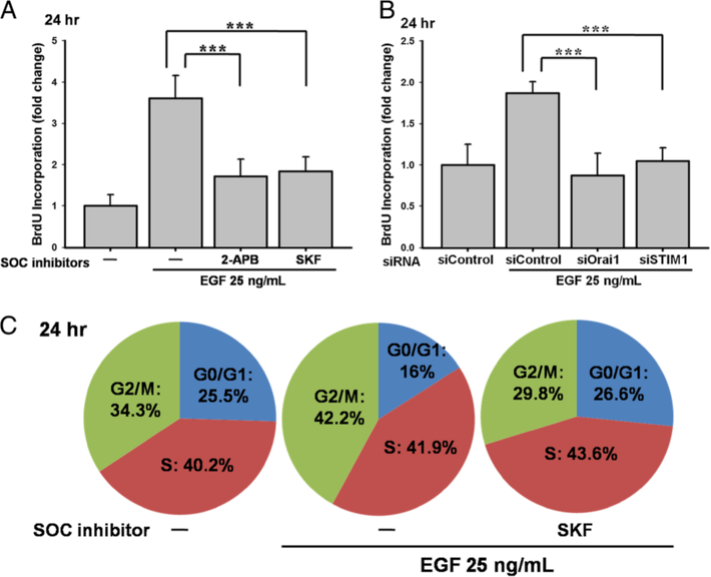
**Effects of SOC inhibitors and knockdown of Orai1**/**STIM1 in BrdU proliferation and cell**-**cycle progression.** (**A**) ARPE-19 cells were pre-treated by 100 μM 2-APB or 20 μM SKF96365 for 30 min. Cells were applied by 25 ng/mL EGF for 24 h. BrdU incorporation assay was used. (**B**) ARPE-19 cells were transfected with Orai1 and STIM1 siRNA. The 25 ng/mL EGF-induced BrdU incorporation was detected. (**C**) The cell-cycle assessed by flow cytometry showed that 25 ng/mL EGF incubation promoted the cell-cycle progression by increasing G2/M percentage, and the 20 μM SKF96365 arrested the cell-cycle in the G0/G1 phase.

To examine the role of STIM1 and Orai1 in EGF-mediated DNA synthesis and cell-cycle progression, the cell proliferation ELISA-based BrdU incorporation assay was used to quantify DNA synthesis in the replicating cells, and flow cytometry was performed to analyze the cell-cycle progression. BrdU incorporation was significantly reduced following treatment with 100 μM 2-APB or 20 μM SKF96365 (both *** p < 0.001; Figure 6A) and by knockdown of Orai1 or STIM1 (both *** p < 0.001; Figure 6B). As shown in Figure 6C, cell cycle arrested in the G0/G1 phase in the presence of 20 μM SKF96365 (16 % (EGF) vs. 26.6% (EGF after SKF pre-treatment)).

### Mitogen-activated protein kinase kinase (MEK)/ERK 1/2 pathway is involved in EGF-mediated cell proliferation and migration

**Figure 7:**
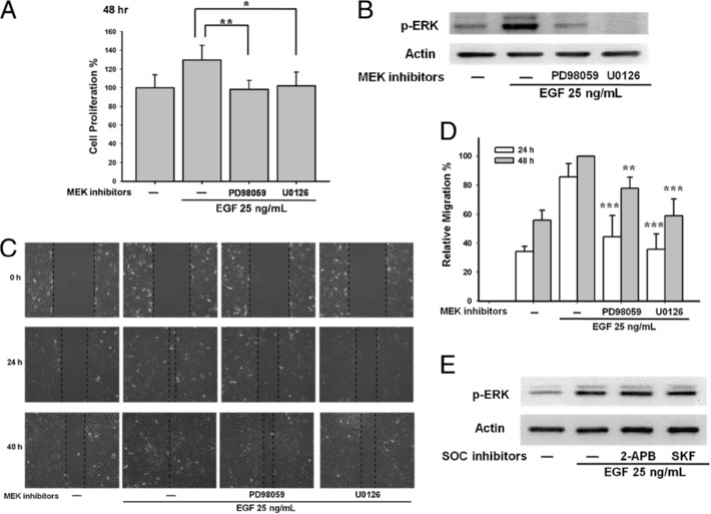
**EGF induced ARPE**-**19 proliferation and migration via MEK**/**ERK 1**/**2 pathway**, **independent of SOC channel pathway.** (**A**) ARPE-19 cells were pre-treated with 20 μM PD98059 or 10 μM U0126 for 30 min and were stimulated by 25 ng/mL EGF for 48 h. WST-1 assay was performed. (**B**) Both 20 μM PD98059 and 10 μM U0126 efficiently reduced the ERK 1/2 phosphorylation in ARPE-19 cells evoked by 25 ng/mL EGF. (**C**) ARPE-19 cells were applied with 20 μM PD98059 or 10 μM U0126 for 30 min followed by 25 ng/mL EGF for 24 h and 48 h. Migration assay was performed. (**D**) Quantitative analysis of cell migration of Figure 7C. (**E**) Cells were applied by 100 μM 2-APB and 20 μM SKF96365, EGF-induced ERK 1/2 phosphorylation was determined by Western blots.

The MEK/ERK 1/2 pathway is an important pathway in proliferation. Pretreatment with MEK inhibitors 20 μM PD98059 and 10 μM U0126 reduced EGF-mediated ARPE-19 proliferation (**p < 0.01 and *p < 0.05, respectively; Figure 7A). Importantly, EGF evoked a strong phosphorylation of ERK 1/2 that was suppressed by the MEK inhibitors PD98059 and U0126 (Figure 7B). Furthermore, pre-treatment with 20 μM PD98059 and 10 μM U0126 reduced the EGF-induced ARPE-19 cell migration (Figure 7C and 7D). However, pre-treatment with the SOC channel inhibitors 100 μM 2-APB and 20 μM SKF96365 had no effect on ERK 1/2 phosphorylation (Figure 7E), thereby indicating that the ERK 1/2 phosphorylation was independent of SOC channel signaling.

### Phosphatidylinositol 3 kinases (PI3K)/Akt pathway is involved in EGF-mediated cell proliferation and migration in ARPE-19 cells

**Figure 8:**
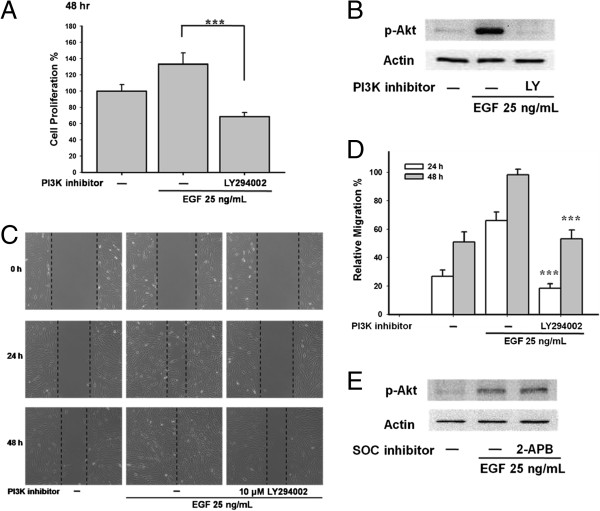
**PI3K**/**Akt pathway is involved in EGF**-**mediated ARPE**-**19 proliferation and migration**, **independent of SOC regulation.** (**A**) ARPE-19 cells were pre-treated with the PI3K inhibitor 10 μM LY294002 for 30 min followed by 25 ng/mL EGF treatment 48 h. Cell proliferation was detected by WST-1 assay. (**B**) Cells were applied by 10 μM LY294002. EGF-evoked phosphorylation of Akt was detected by Western blots. (**C**) ARPE-19 cells were pre-treated by 10 μM LY294002 for 30 min and were stimulated by 25 ng/mL EGF. Cell migration assay was performed. (**D**) Quantitative analysis of cell migration of Figure 8C. (**E**) Cells were pre-treated by 100 μM 2-APB. EGF-induced Akt phosphorylation was examined by Western blots.

The PI3K/Akt pathway is also an important EGF-mediated cell proliferation pathway. Pretreatment with the PI3K inhibitor LY294002 (10 μM) reduced EGF-mediated cell proliferation (*** p < 0.001; Figure 8A). Importantly, 10 μM LY294002 inhibited phosphorylation of Akt (Figure 8B) and EGF-mediated cell migration (Figure 8C and 8D). However, the SOC channel inhibitor 100 μM 2-APB did not suppress EGF-activated Akt phosphorylation (Figure 8E), indicating that Akt phosphorylation was not regulated by SOC channels.

### Knocking down Orai1 and STIM1 suppressed EGF-mediated cell proliferation and migration, but not via suppressing ERK 1/2 or Akt phosphorylation

**Figure 9:**
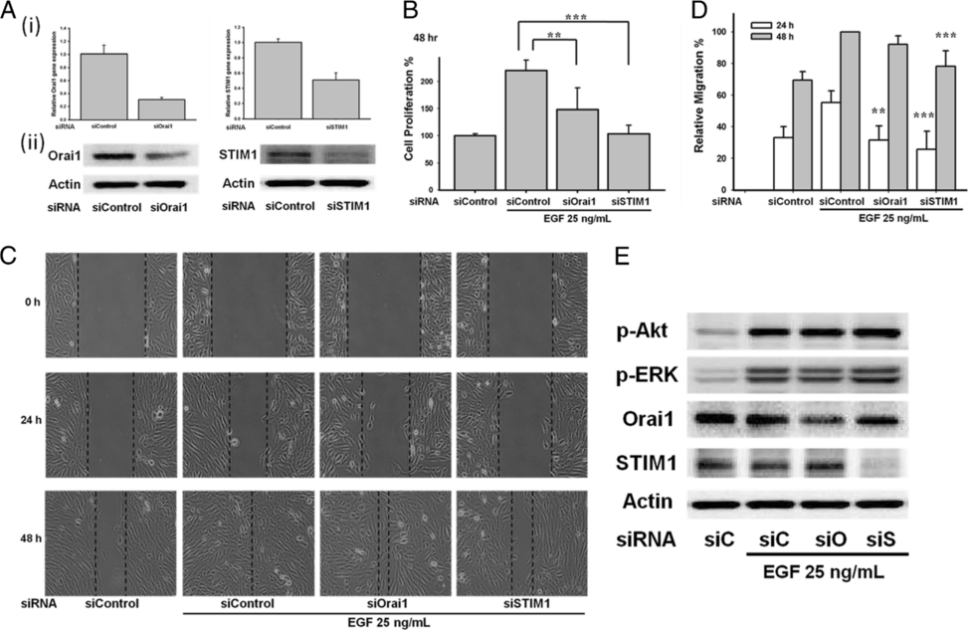
**Involvement of Orai1 and STIM1 in EGF**-**mediated cell proliferation and migration.** (**A i**, **ii**) ARPE-19 cells were transfected with another pair of Orai1 and STIM1 siRNA. Expression of Orai1 and STIM1 was detected by real-time PCR (**i**) and Western blots (**ii**). (**B**-**C**) ARPE-19 cells were transfected with Orai1 and STIM1 siRNA. WST-1 assay or cell migration assay was used. (**D**) Quantitative analysis of ARPE-19 cell migration of Figure 9C. (**E**) ARPE-19 cells were transfected with Orai1 and STIM1 siRNA. EGF-mediated phosphorylation of ERK 1/2 and Akt were detected by Western blots.

To strengthen the role of STIM1/Orai1 signaling in ARPE-19 cells, another pair of Orai1 siRNA and STIM1 siRNA was transfected into the ARPE-19 cells. The Orai1 and STIM1 siRNAs reduced expression of RNA (Figure 9A(i)) and protein (Figure 9A(ii)). Knocking down Orai1 and STIM1 suppressed cell proliferation (**p < 0.01 and ***p < 0.001, respectively; Figure 9B) and migration (Figure 9C and 9D). To clarify the cross-talk signaling between STIM1/Orai1 and ERK 1/2 or Akt, the EGF-mediated ERK 1/2 and Akt phosphorylation were tested after transfection with Orai1 siRNA and STIM1 siRNA. The knockdown of Orai1 and STIM1 did not alter the EGF-evoked ERK 1/2 or Akt phosphorylation in ARPE-19 cells (Figure 9E).

## Discussion

The results of the present study demonstrated that EGF could trigger cell proliferation and migration via STIM1, Orai1, and phosphorylation of ERK 1/2 and Akt. In RPE cells, the secretion of VEGF is regulated by calcium entry through voltage-dependent L-type calcium channels [16]. Transient receptor potential cation (TRPC) channels have been reported to involve in the maintenance of basal cellular processes, such as basal secretion of cytokines [17]. In corneal epithelial cells, TRPC4-mediated SOC activation is essential in EGF signaling [18,19]. Combination of molecular biological and electrophysiological approaches, Cordeiro and Strauss reported a functional SOC channel composed of Orai and STIM subunits in RPE cells [14].Consistent with the studies by Cordeiro, our study also confirmed the expression of Orai1 and STIM1 in the ARPE-19 cells.

Yang et al. reported that STIM1 and Orai1 regulate the migration and metastasis of breast cancer [20]. In addition, Chen et al. revealed that STIM1-dependent signaling is important for cervical cancer cell growth, migration, and angiogenesis [12]. Subsequently, Yoshida et al. showed that STIM1 knockdown suppressed SOC entry, cell proliferation, and tumorigenicity in A431 cells [21]. In our study, we found that ARPE-19 cell proliferation and migration were suppressed by the SOC channel blockers 2-APB and SKF as well as siRNA against Orai1 or STIM1. The ARPE-19 cells treated with SKF96365 were arrested in the G0/G1 phase. The results are similar to previous studies in which cell proliferation was inhibited by cell cycle arrest in the G0/G1 phases after manipulation of Orai1/STIM1 signaling [22,23].

Using the cell-attached patch-clamp technique, Ma et al. were the first to show that EGF stimulates SOC in both a time-dependent and dose-dependent manner in human glomerular mesangial cells [10]. Chen et al. nicely demonstrated that EGF-induced calcium influx is a STIM1-dependent process that modulates cell growth in cervical cancer cells [12]. Consistent with this, our previous study also observed increased calcium signals evoked by EGF in A431 cancer cells [24]. From the literatures, EGF-mediated intracellular calcium increase is through a variety of channels [25-27]. However, in our study, we did not see typical SOC signals evoked by EGF in ARPE-19 cells (data not shown). Since the involvement of STIM1 and Orai1 in EGF-mediated cell growth is strongly supported by our study results, we attribute our lack of typical SOC signals to the complex pattern of activation of various calcium channels induced by EGF in ARPE-19 cells.

Phosphorylation of ERK 1/2 and Akt are involved in cell proliferation [28,29]. Our studies demonstrated that inhibition of ERK 1/2 phosphorylation by PD98059 and U0126, or inhibition of Akt phosphorylation by LY294002, suppressed RPE cell proliferation/migration. These findings are consistent with studies by Defoe and colleagues [30] that revealed the importance of PI3K and MAPK pathways in EGF signaling in RPE cells. We also found that SOC channel inhibitors or knockdown of Orai1 and STIM1 blocked cell proliferation and migration, but did not influence the phosphorylation levels of ERK 1/2 and Akt. The regulation of RPE cell proliferation/migration by EGF remains unclear. Our results indicated that, at least, two distinct proliferative pathways were regulated by EGF, which control cellular responses.

PVR is the most common complication in patients recovering from retinal detachment surgery [31]. The molecular mechanisms underlying the development of PVR are still elusive. The involvement of EGF-mediated proliferative pathways in the cellular processes of PVR has been widely reported [30,32]. Liang et al. indicated that glucosamine (GlcN) might be useful in the treatment of EGF-mediated ocular proliferative disorders [33]. These observations in combination with those of the present study imply that STIM1/Orai1, MEK/ERK 1/2 and PI3K/Akt pathways are important mediators of PVR. More studies are needed to determine the molecular basis and pathogenesis of PVR in culture cells as well as animal models.

## Conclusions

**Figure 10:**
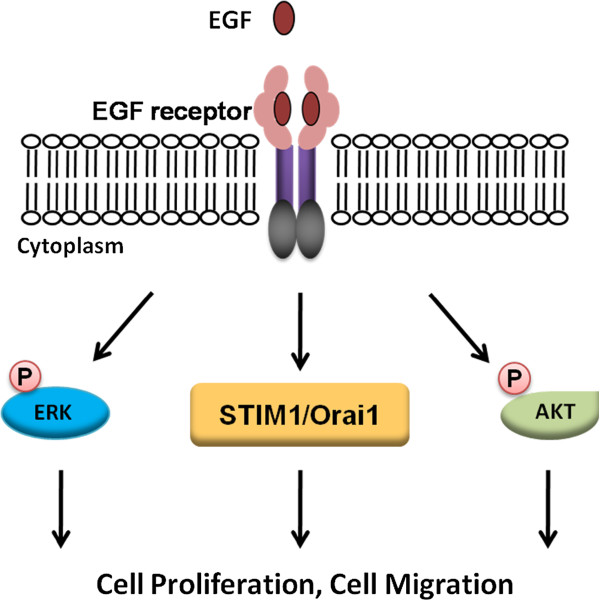
**Schematic representation of EGF**-**mediated ARPE**-**19 cell proliferation and migration.** Functional STIM1, Orai1, and phosphorylation of ERK 1/2 and Akt, are essential in EGF-induced RPE cell proliferation and migration.

Our results highlight the importance of STIM1, Orai1, ERK 1/2 and Akt in EGF-mediated proliferative pathways in ARPE-19 cells (Figure 10). EGF plays a key role in the development of PVR. Our studies revealed that STIM1, Orai1, and phosphorylation of ERK 1/2 and Akt, may serve as potential therapeutic targets for future clinical management of PVR.

## Abbreviations

RPE: Retinal pigment epithelium; PVR: Proliferative vitreoretinopathy; EGF: Epidermal growth factor; SOC: Store-operated calcium; STIM1: Stromal interaction molecule 1.

## Competing interests

The authors declare that they have no competing interests.

## Authors’ contributions

Conceived and designed the experiments: IY and WC*. Performed the experiments: IY, YT, SC and HL. Analyzed the data: IY, YT, SC, WH and WC*. Contributed reagents/materials/analysis tools: IY, LL, MH, BC and WC*. Wrote the paper: IY and WC*. All authors read and approved the final manuscript.
